# Complexity of the prey spectrum of *Agaronia propatula* (Caenogastropoda: Olividae), a dominant predator in sandy beach ecosystems of Pacific Central America

**DOI:** 10.7717/peerj.4714

**Published:** 2018-04-30

**Authors:** Nathan J. Robinson, Winfried S. Peters

**Affiliations:** 1 Cape Eleuthera Island School, Cape Eleuthera Institute, Eleuthera, The Bahamas; 2 Goldring-Gund Marine Biology Station, Playa Grande, Santa Cruz, Guanacaste, Costa Rica; 3 School of Biological Sciences, Washington State University, Pullman, WA, USA

**Keywords:** *Agaronia*, Ontogenetic niche shift, Trophic network, Predator–prey interaction, Sandy beach, Olividae, Cannibalism, Tropical Eastern Pacific

## Abstract

Olivid gastropods of the genus *Agaronia* are dominant predators within invertebrate communities on sandy beaches throughout Pacific Central America. At Playa Grande, on the Pacific Coast of Costa Rica, we observed 327 natural predation events by *Agaronia propatula*. For each predation event, we documented prey taxa and body size of both predator and prey. The relationship between predator and prey size differed for each of the four main prey taxa: bivalves, crustaceans, heterospecific gastropods, and conspecific gastropods (representing cannibalism). For bivalve prey, there was increased variance in prey size with increasing predator size. Crustaceans were likely subdued only if injured or otherwise incapacitated. Heterospecific gastropods (mostly *Olivella semistriata*) constituted half of all prey items, but were only captured by small and intermediately sized *A. propatula*. Large *O. semistriata* appeared capable of avoiding predation by *A. propatula*. Cannibalism was more prevalent among large *A. propatula* than previously estimated. Our findings suggested ontogenetic niche shifts in *A. propatula* and a significant role of cannibalism in its population dynamics. Also indicated were size-dependent defensive behavior in some prey taxa and a dynamic, fine-scale zonation of the beach. The unexpected complexity of the trophic relations of *A. propatula* was only revealed though analysis of individual predation events. This highlights the need for detailed investigations into the trophic ecology of marine invertebrates to understand the factors driving ecosystem structuring in sandy beaches.

## Introduction

Beaches on macrotidal coasts are highly dynamic habitats controlled by physical factors (McLachlan & Brown, 2006). Inhabitants of sandy beaches, and especially those found in the intertidal zone, show specific morphological, physiological, and behavioral adaptations to the range of physical forces that dominate their ecosystem (Newell, 1979). Moreover, beaches worldwide are under immense pressure due to development, tourism, sea-level changes, and pollution of the coastal environment (Defeo et al., 2009; Schlacher et al., 2008; Pilkey et al., 2011). Studying sandy beach ecosystems therefore does not only promote our knowledge of fundamental biological processes, but can also provide the foundation for more efficient conservation measures.

Gastropods of the genus *Agaronia* in the family Olividae are common on sandy beaches throughout Pacific Central America (López, Montoya & López, 1988; see the taxonomic note below), where they regularly prey on another common olivid, the filter-feeding *Olivella semistriata* (Gray 1839) (Cyrus et al., 2012). *Agaronia* is closely related to the more thoroughly studied genus *Oliva. Oliva* spp. live in soft sediments, most of them in the subtidal zone and adjacent shallow waters (Tursch & Greifeneder, 2001). They are known to hunt by cruising on the substrate (Zeigler & Porreca, 1969), making up for their lack of long-distance sensory capacities by speed of locomotion (Kantor & Tursch, 2001). When they encounter a potential prey item, it is seized by the anterior part of the foot and transferred to the ventral side of the metapodium (posterior foot). The metapodium then closes like a drawstring bag to secure the prey in the so-called metapodial pouch. Finally, the snail burrows into the sediment to consume its prey (Marcus & Marcus, 1959; Olsson & Crovo, 1968; Zeigler & Porreca, 1969; Kantor & Tursch, 2001).

Similar behavior is exhibited by other large olivids including *Olivancillaria auricularia* (Lamarck 1811) (as *O. vesica auricularia*; Rocha-Barreira, 2002) and *Agaronia propatula* (Conrad 1849) (Rupert & Peters, 2011). Unlike *Oliva* spp., which are predominantly active at night, *A. propatula* forages on the intertidal plains of sandy beaches during intermediate and low tide at daylight. Thus, *A. propatula* are more readily observed in the wild (videos showing *A. propatula* capturing prey are available as supplementary materials with Rupert & Peters, 2011, and Cyrus et al., 2012). The gastropod also shows a range of interesting adaptations. For example, it exploits wave action to move rapidly using its expanded foot as an underwater sail, and has a pronounced capability of sensing movement and vibrations in the sediment to detect potential prey (Cyrus et al., 2012). It autotomizes the part of its foot that is used to form the pouch in which prey is stored (Rupert & Peters, 2011), but previous studies have failed to identify the natural trigger of autotomy in *A. propatula* (Cyrus & Peters, 2014; Cyrus et al., 2015). The species also exhibits size-dependent cannibalism, resulting in different roles of different size classes of the population in the trophic network of the beach ecosystem (Cyrus et al., 2015). Considering these facts, *A. propatula* appears to be an easily accessible model species for the study of the behavioral and sensory basis of complex trophic interactions.

Prey spectra of *A. propatula* have already been reported for two Central American locations (Cyrus et al., 2012). In this previous study, various potential prey items were also presented to foraging individuals. Of all the prey tested, only sand dollars were rejected, leading to the conclusion that *A. propatula* was an opportunistic predator whose prey spectrum mirrored the composition of the local community, with the exception of echinoderms (Cyrus et al., 2012). As a test of this hypothesis, we conducted a more detailed characterization of *A. propatula*’s prey spectrum. This new study analyzed almost four times as many predation events and, more importantly, measurements of individual predator and prey sizes. In the context of the recent recognition of the importance of individual variation and ontogenetic niche shifts in population ecology (Petchey et al., 2008; Bolnick et al., 2011; Violle et al., 2012; Hart, Schreiber & Levine, 2016), it has been suggested that meaningful quantitative conclusions regarding predator–prey relationships can only be based on individual interaction data (Trebilco et al., 2013; Nakazawa, 2017). Here, we evaluate such individual data for *A. propatula* operating in its natural environment, revealing previously unknown layers of complexity in the species’ trophic relationships.

## Methods

### Taxonomic note

The taxonomy within the Olividae is notoriously problematic (Tursch & Greifeneder, 2001; Kantor et al., 2017). The widely distributed and very abundant *O. semistriata* (Gray 1839), for example, has consistently been confused in the recent literature with the equally common but more southerly distributed *O. columellaris* (Sowerby 1825), as reviewed by Troost et al. (2012).

In the case of the Central American *Agaronia* spp., we have often found the existing identification literature (Keen, 1971; López, Montoya & López, 1988; Sterba, 2004) to be ambiguous and not always helpful in the field. Following previous work (Rupert & Peters, 2011; Cyrus et al., 2012, 2015; Cyrus & Peters, 2014), we refer to the predatory *Agaronia* sp. observed in this study as *A. propatula* (Conrad 1849), albeit with hesitation.

### Field observations and data analysis

**Figure 1:**
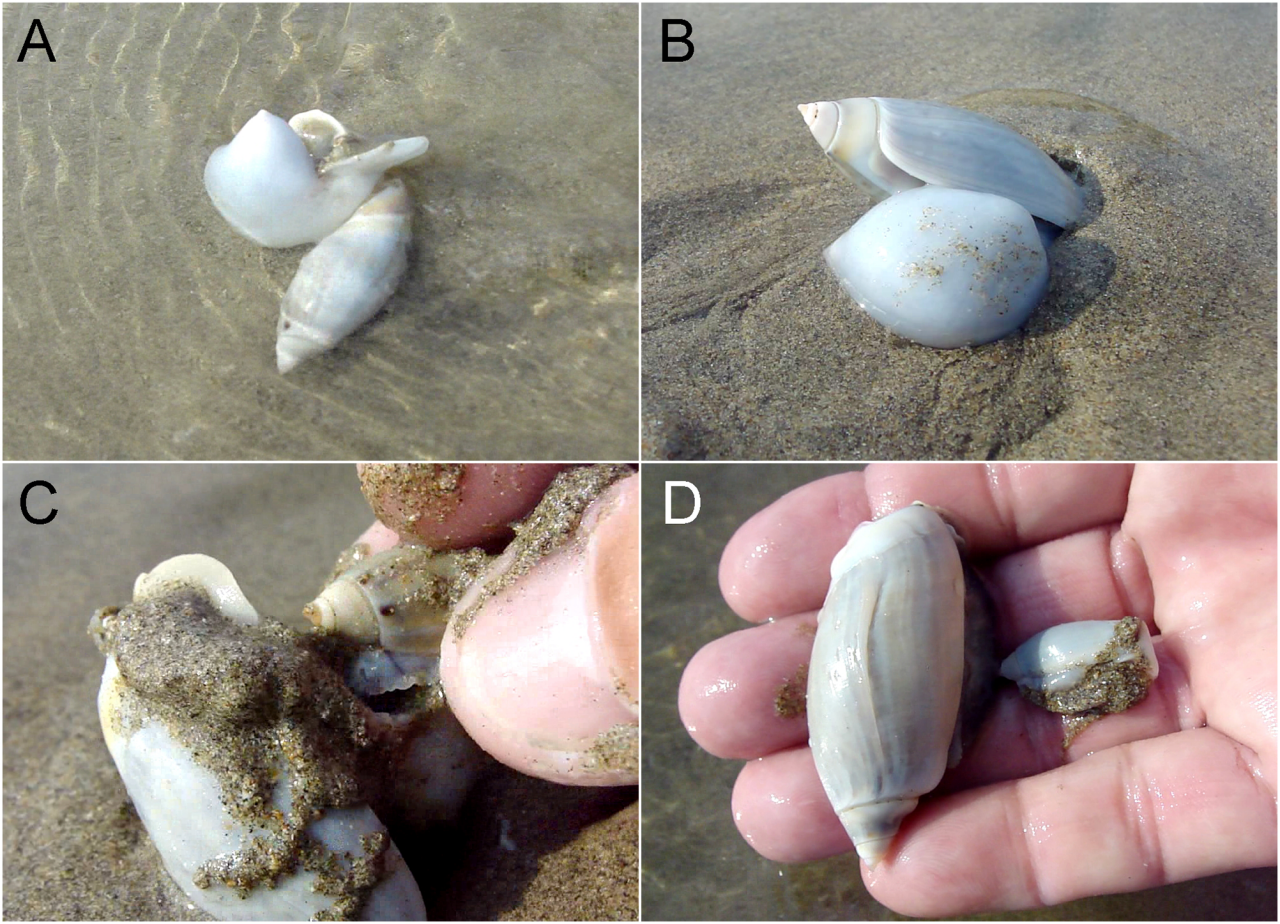
Example of a predation event recorded in the wild. A large *Agaronia propatula* with filled metapodial pouch is observed sailing with the backwash (A) before burrowing into the sediment (B). The snail is captured and the prey item removed from the pouch (C); it is a smaller conspecific (D). Photo credit: Winfried S. Peters.

Between November 2011 and April 2017, the intertidal zone of Playa Grande, Costa Rica (10°20′N, 85°51′W), was patrolled during opportunistic trips to search for *A. propatula* with filled metapodial pouches. Searches were performed during daylight, usually within the six hours around the time of low tide. Filled pouches almost always contained a prey item, indicating a successful predation attempt. Successful predators were picked up while burrowing into the sediment or sailing seaward in the backwash, and the prey was released by gently pressuring the metapodial pouch between fingertips (Fig. 1). The size of the predator and its prey were measured to the nearest 0.1 mm either using calipers or on images taken of predator and prey next to a ruler using ImageJ (https://imagej.nih.gov/ij/). Size was defined in gastropods as the shell length from apex to base, in bivalves as the longest axis across the shell, and in crustaceans as the anterior–posterior body length. All animals were released immediately after being measured or photographed at the same location where they had been collected. The handling period between capture and release was shorter than two minutes. A small number of predation events involving mole crabs, *Emerita* sp., which were observed on the beach of El Cuco, El Salvador (13°10′N, 88°06′W), in May 2011, were included in our analyzes for comparative purposes, as specified in the Results section.

The original data is available online as Table S1; Table 1 gives an overview. When the coefficient of determination (*r*^2^) suggested a possible correlation between predator size and prey size, the relationship was described by the geometric mean functional relationship (also known as standardized major axis) following Draper & Smith (1998). This was necessary since neither of the two parameters (predator size and prey size) could be considered independent variables (i.e., free of significant statistical error); in such cases conventional regression analysis does not yield meaningful descriptions of the relationship between the parameters. Binomial probability calculations and *χ*^2^ tests were performed using online tools available at http://vassarstats.net/.

**Table 1:** Prey taxa found in metapodial pouches of *Agaronia propatula* at Playa Grande.

Prey taxon	Number of occurrences (% of the total)
Gastropoda	165 (50.5)
*Olivella semistriata*	163 (49.8)
*Mazatlania fulgurata*	2 (0.6)
Cannibalism	31 (9.5)
Bivalvia	88 (26.9)
*Donax* sp.	69 (21.2)
*Pitar* sp.	5 (1.5)
*Tivela* sp.	1 (0.3)
*Milbe* sp.	1 (0.3)
Bivalvia indet.	12 (3.7)
Crustacea	43 (13.1)
*Emerita* sp.	26 (7.9)
Crustaceae indet.	17 (5.2)
Total	327 (100.0)

In a subset of the documented predation events, shelled prey items (21 *O. semistriata*, 14 *Donax* sp.) that had been removed from pouches were placed in small tanks containing sand and water immediately. Any activities of the released prey were recorded over the following 5 min.

Field studies were performed under the research permits ACT-OR-D-015 and ACT-OR-DR-064 to W.S.P. from the Ministerio de Ambiente y Energia de Costa Rica.

### Modeling of expected rates of cannibalism

The expected distribution of successful cannibalization events between the different size classes of *A. propatula* was calculated numerically from the empirical size distribution of successful hunters under the assumption that individuals meet randomly in their habitat. Conditions for successful cannibalistic interactions empirically defined by Cyrus et al. (2015) were applied, as detailed in the Discussion section. The calculations are available online as Table S2.

## Results

### Predator size distribution

**Figure 2:**
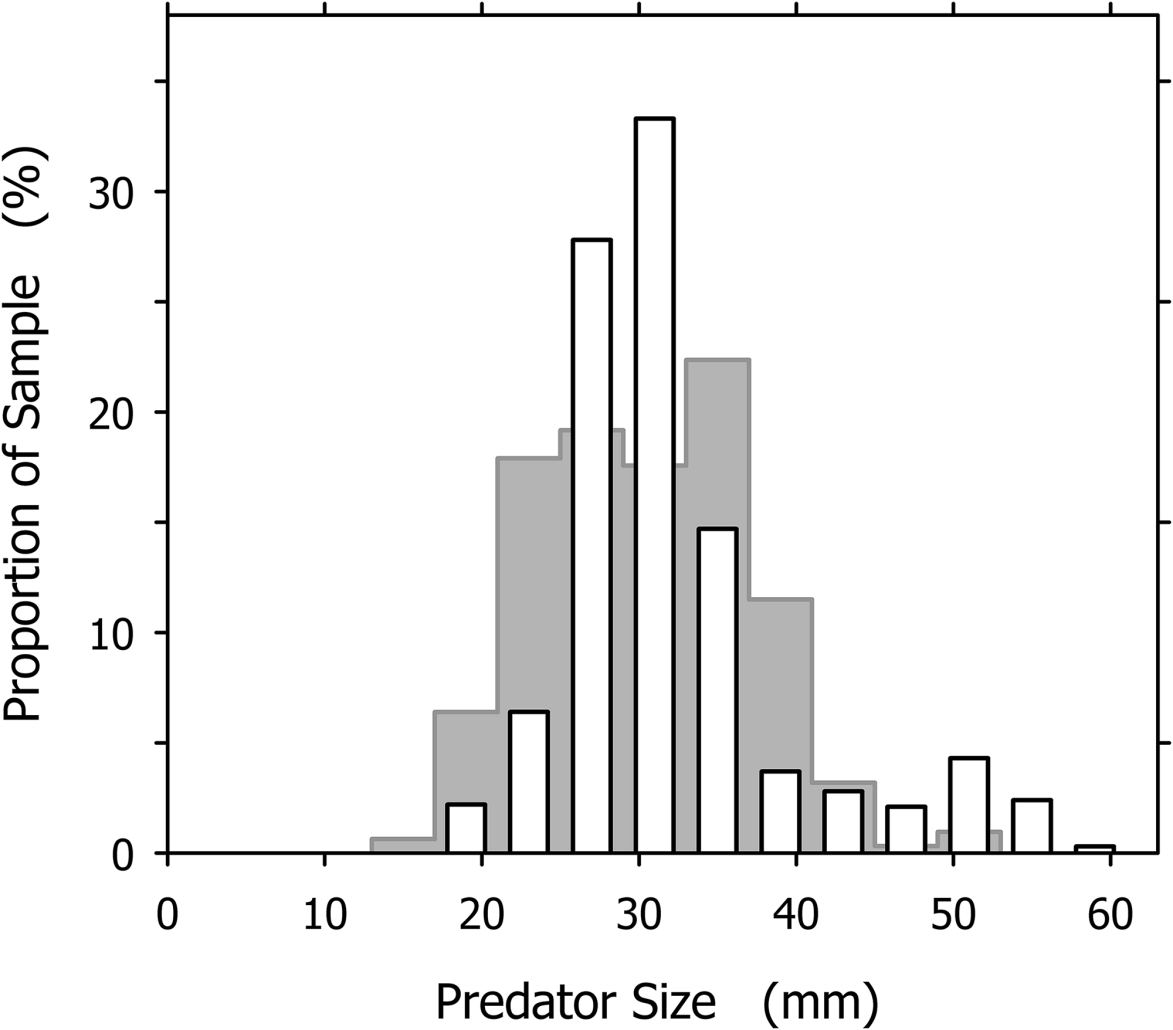
Size distribution of *Agaronia propatula* (4 mm intervals from 17–21 to 57–61 mm) found with prey in their metapodial pouches at Playa Grande (*n* = 327; white bars). For comparison, the size distribution of actively foraging *A. propatula* on the intertidal sediment surface at the same location reported by Cyrus et al. (2015) is shown (gray bars in background; *n* = 313).

The largest and smallest *A. propatula* we ever measured on Playa Grande had shell lengths of 60.1 and 13.2 mm, respectively, but such extreme sizes were rare. The subset of 327 *A. propatula* found with prey in their pouches, which is analyzed in the present study, ranged from 18.0 to 57.4 mm, with intermediate sizes from 25 to 37 mm representing a large majority (76%; Fig. 2). The size distribution of successful predators resembled a previously published distribution of *A. propatula* foraging in the intertidal zone at Playa Grande (Cyrus et al., 2015), although the peak at intermediate sizes seemed narrower and higher in the distribution of successful predators (Fig. 2).

### Metapodial pouch contents

Metapodial pouches never contained more than one prey item. About half of the prey found in pouches were *O. semistriata* (Table 1). Only in two cases was another heterospecific gastropod found, *Mazatlania fulgurata* (Philippi 1846). Over one-quarter of the prey were bivalves, with the genus *Donax* (burrowing clams) dominating. Crustaceans (mostly mole or sand crabs, *Emerita* sp.) and conspecifics contributed the rest of the prey spectrum (Table 1). Several common inhabitants of the study beach were not recorded as prey, including lunulate sand dollars (*Mellita* spp.), flatworms (Plathyhelminthes), and polychaetes.

Many shelled prey released from metapodial pouches were alive. We placed 21 *O. semistriata* and 14 *Donax* sp. in small tanks immediately after they were removed from pouches. After 5 min, 19 (90%) of the gastropods and 11 (79%) of the bivalves had resumed normal activity such as crawling or burrowing. In contrast, almost all of the crustaceans found in pouches were dead or so heavily injured that they had lost the ability for controlled locomotion. At least 12 of the 43 crustaceans removed from pouches had body parts missing or were visibly damaged.

Pouches often contained sand in addition to the prey item, especially when the prey was small (<10 mm). Occasionally pouches would be found that only contained sand with no identifiable prey. These cases were not regarded successful predation attempts and therefore are not listed in Table 1.

**Figure 3:**
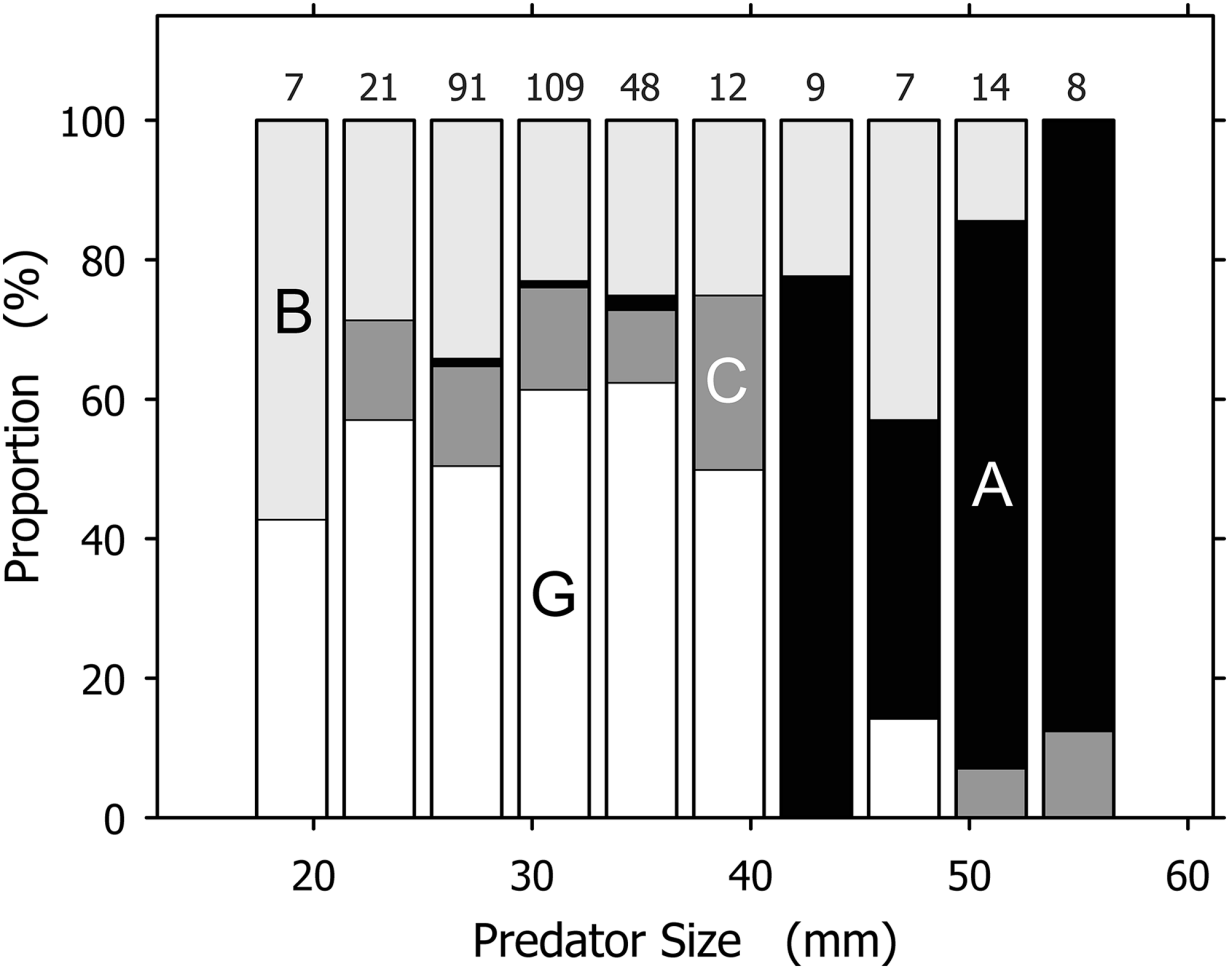
Prey spectra for size classes of *Agaronia propatula*. Prey was categorized as B, bivalves (light gray); G, gastropods (of which over 98% were *Olivella semistriata*; white); C, crustaceans (dark gray); and A, *A. propatula* (cannibalism; black). The largest size class (57–61 mm) is omitted since it included only one case, a bivalve. Sample sizes are given on top of the columns.

Although useful as an overview, the total prey spectrum presented in Table 1 failed to convey the complexity revealed by our data. Prey spectra for individual *A. propatula* size classes spanning 4 mm of shell length indicated significant size-dependent shifts (Fig. 3). The most common prey for small (<21 mm), intermediate (21–41 mm), and large (>41 mm) size classes of *A. propatula* were bivalves, hetrospecific gastropods, and conspecifics, respectively. Below, the relationship between predator size and prey size is analyzed for each major prey taxon.

### Bivalve prey

**Figure 4:**
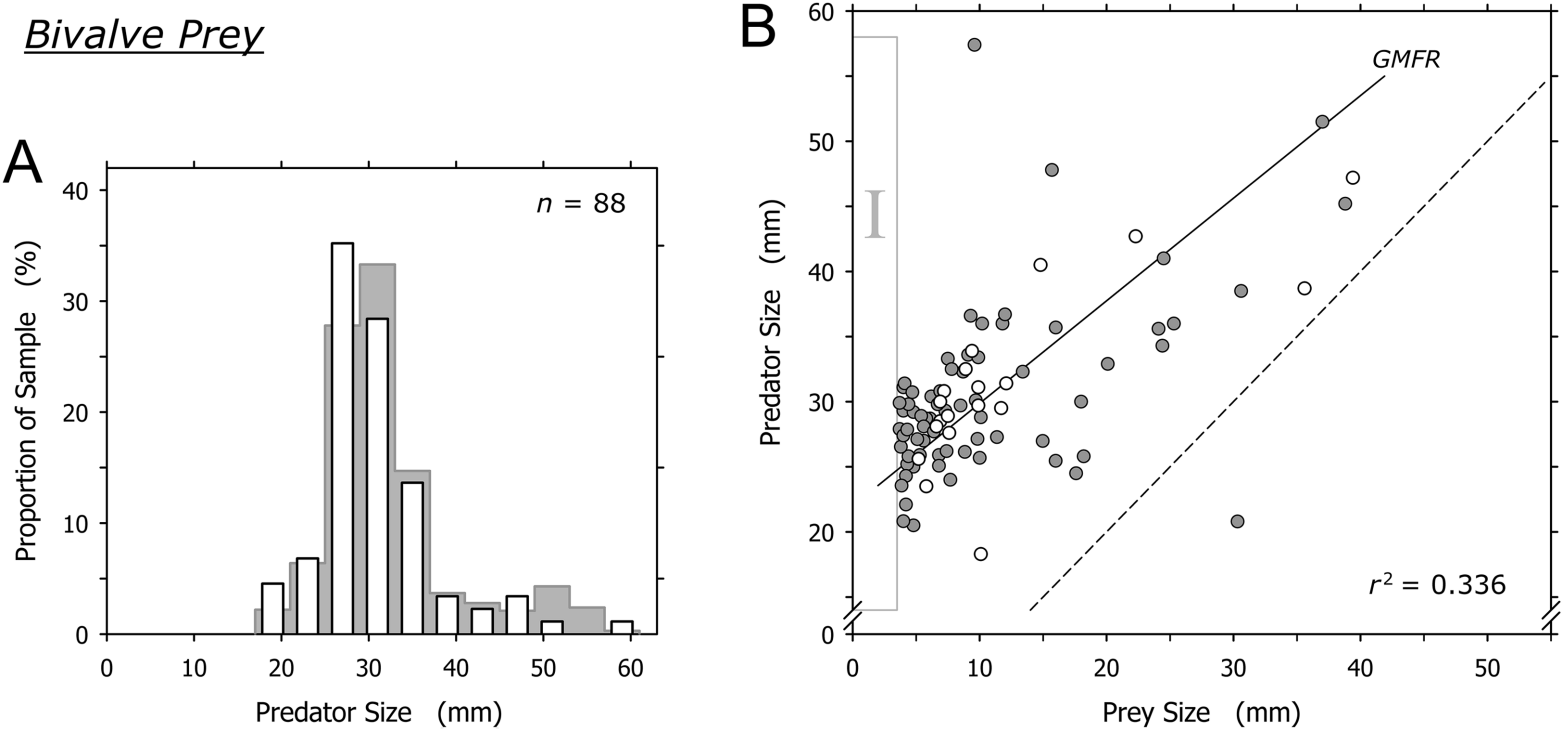
Relationship between shell lengths of *Agaronia propatula* and its bivalve prey. (A) Size distribution of *A. propatula* with bivalves in their metapodial pouches (white bars). The size distribution of *A. propatula* carrying prey of any taxon is shown in the background. (B) Predator size plotted vs prey size. Gray circles represent predation on *Donax* sp., white circles represent predation on other bivalves. The coefficient of determination (*r*^2^) and the geometric mean functional relationship (GMFR) for the entire bivalve dataset are shown. The dashed line indicates a predator-to-prey size ratio of 1; interactions in which the prey was larger than the predator lie to the right of this line. Here, as well as in Figs. 5 and 6, zones of interest are marked by Roman numerals to facilitate discussion in the main text.

Bivalves were captured by *A. propatula* of all sizes (Fig. 4A). The largest predated bivalves (*Pitar* sp., 39.4 mm; *Donax* sp., 38.8 mm) were similar in size to the largest non-predated bivalve (*Donax* sp., 42 mm) that we ever observed alive on Playa Grande (Fig. 4B). There was a trend for larger *A. propatula* to capture larger bivalves (*r*^2^ = 0.336; Fig. 4B). In fact, the shell length of bivalve prey often was similar, and in one case even larger than that of the gastropods that had captured it (Fig. 4B). At the other end of the size spectrum, bivalves of under 3.7 mm shell length were notably absent (see the field marked “I” in Fig. 4B). This is interesting as bivalve prey immediately above this size limit were found frequently; one-fifth of all captured bivalves were between 3.7 and 4.7 mm.

### Crustacean prey

**Figure 5:**
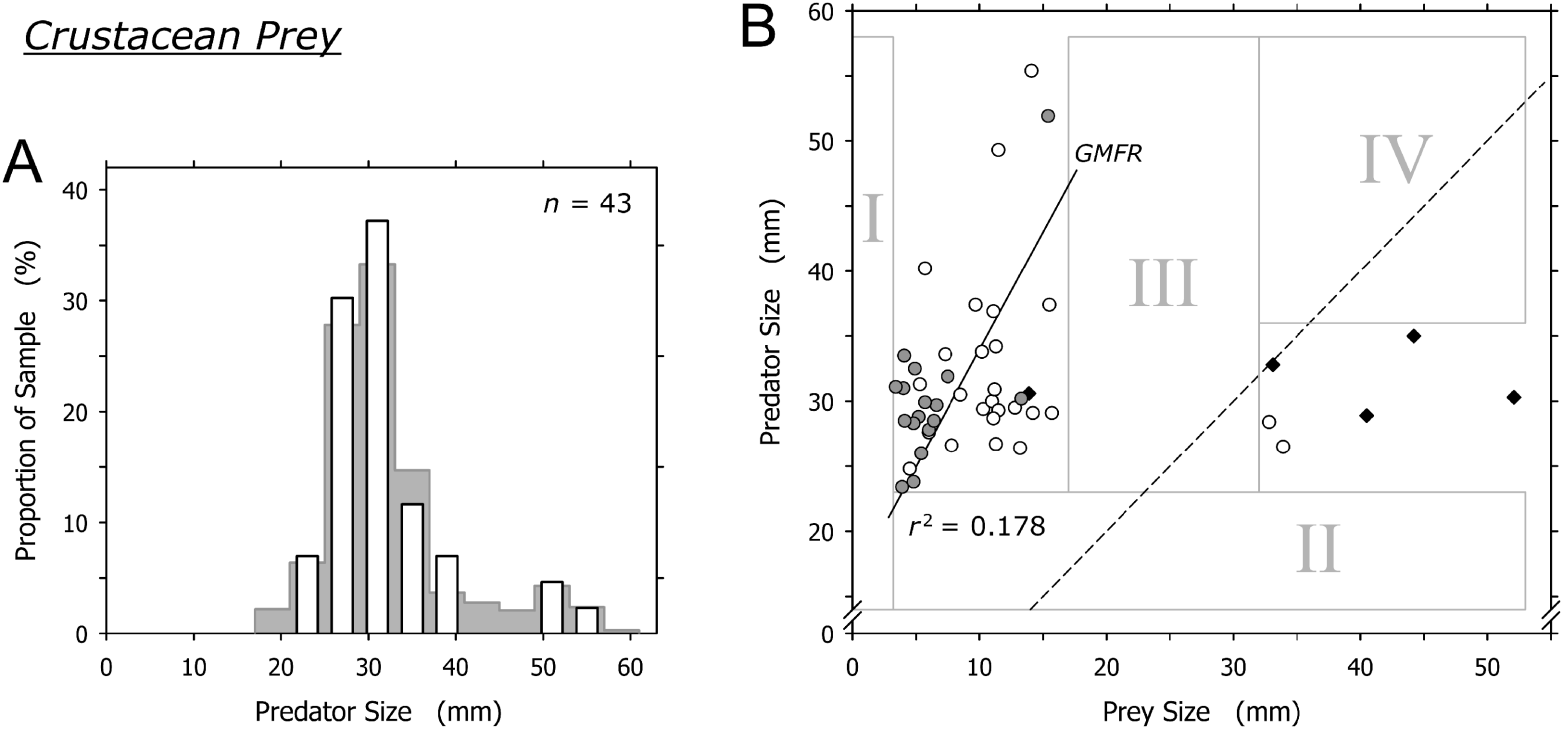
Relationship between shell length of *Agaronia propatula* and body length of its crustacean prey. (A) Size distribution of *A. propatula* with captured crustaceans (white bars); the size distribution of *A. propatula* carrying any prey is shown in the background. (B) Plot of predator vs prey size. Predation events observed at Playa Grande are given as circles; white circles indicate predation on *Emerita* sp., while gray ones represent predation on other crustaceans. *r*^2^ for the subset of observations from Playa Grande with prey sizes below 20 mm and the corresponding GMFR are indicated. Observations of successful predation on *Emerita* sp. made at El Cuco are shown as black diamonds. The dashed line marks the predator-to-prey size ratio of 1.

Crustaceans were captured by most size classes of *A. propatula* (Fig. 5A). Similar to bivalve prey, there was a sharp lower limit of prey size at just below 4 mm (field I in Fig. 5B). There also was a lower limit of predator size at about 23 mm (note lack of data in field II in Fig. 5B). While most prey items ranged between 6 and 15 mm, two prey items exceeded 32 mm and were bigger than their prey. Intermediately sized prey was lacking (no data in field III; Fig. 5B). Consequently, there was no correlation between predator and prey size when the entire dataset was considered (*r*^2^ = 0.016), while the analysis of the subset of events involving small prey only (<20 mm) hinted at a possible trend for larger predators to capture larger prey (*r*^2^ = 0.178; Fig. 5B).

The two isolated cases where prey exceeded 30 mm involved mature *Emerita* sp.; one carried an egg mass under the abdomen. A similar pattern of size ratios between *A. propatula* and their *Emerita* sp. prey was found on the beach at El Cuco in El Salvador (black diamonds in Fig. 5B). Combined, the two datasets show an isolated group of six *Emerita* that exceeded the shell lengths of their predators (Fig. 5B). Two of the six large *Emerita* were dead, and the others proved incapable of burrowing into the sediment or any kind of locomotion after being removed from the metapodial pouches. All large *Emerita* had been captured by relatively small *A. propatula* (26.5–35.0 mm), whereas larger predators were found with small crustacean prey but never with mature *Emerita* (Fig. 5B; note lack of data in field IV).

### Gastropod prey (excluding cannibalism)

**Figure 6:**
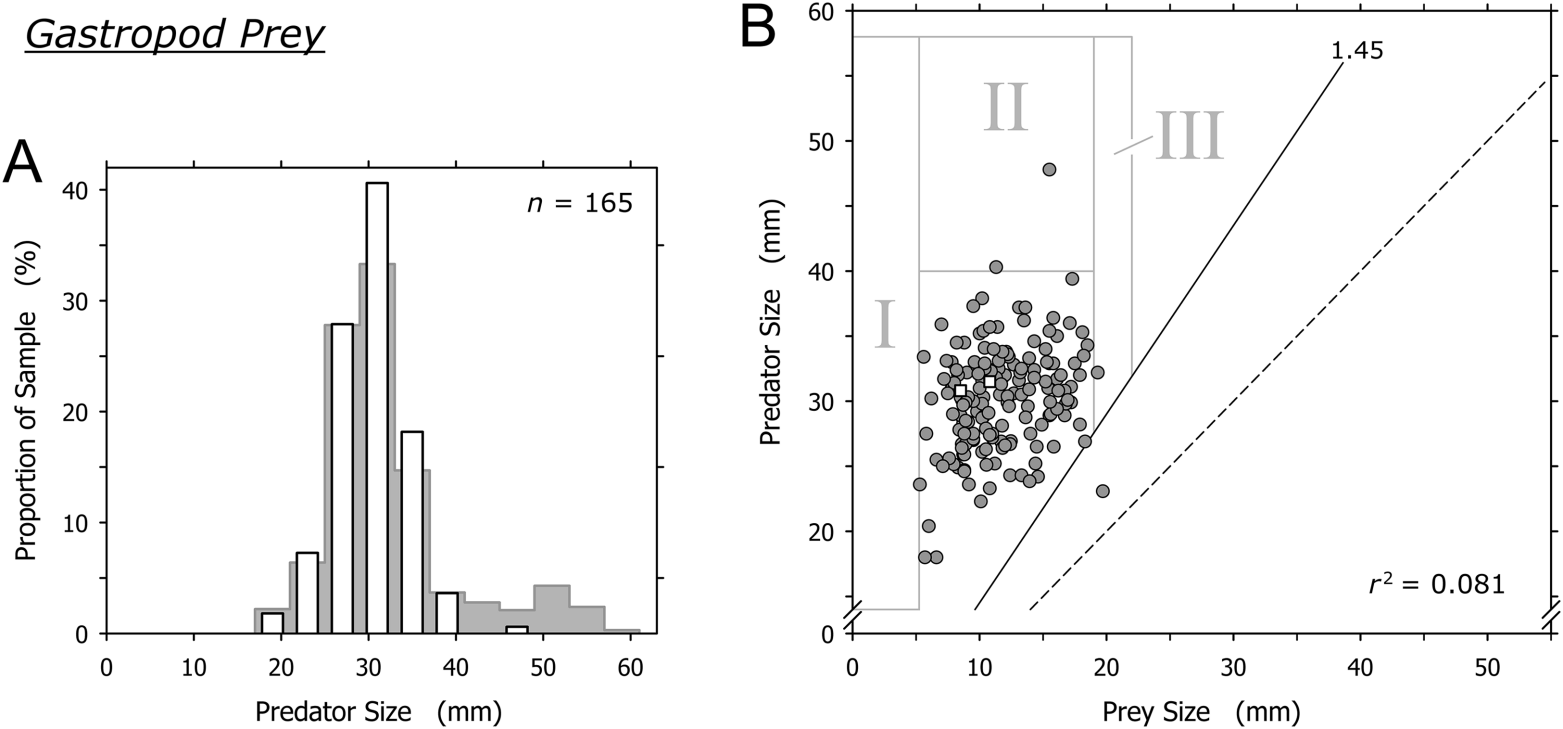
Relationship of the shell lengths of *Agaronia propatula* and its heterospecific gastropod prey. (A) Size distribution of *A. propatula* with other gastropods in their metapodial pouches (white bars). The size distribution of predators carrying any prey is given in the background. (B) Plot of predator size vs prey size. Gray circles and white squares represent predation on *Olivella semistriata* and *Mazatlania fulgurata*, respectively. *r*^2^ is given for the entire gastropod dataset. The predator-to-prey size ratio of 1 (dashed line) and the postulated threshold ratio of 1.45 for predation on gastropods (solid line) are highlighted.

Cannibalism excluded, *O. semistriata* was the only gastropod captured by *A. propatula* at a significant frequency (163 out of 165 observations involving gastropod prey; Table 1). The sizes of predators and of their gastropod prey were not correlated (*r*^2^ = 0.081). Our data was in line with the threshold value of the predator-to-prey size ratio for predation on gastropods postulated by Cyrus et al. (2015): with the exception of a single outlier, the shell length of successful predators always was more than 1.45 times that of their prey (Fig. 6B). As with bivalve and crustacean prey, there was a sharp lower limit of gastropod prey size (field I, Fig. 6B).

Large *A. propatula* seemed unsuccessful in preying on other gastropods, as documented in Fig. 6A and by the paucity of data-points in field II in Fig. 6B. If successful predation attempts on *O. semistriata* were randomly distributed across the observed size spectrum of successful *A. propatula* (Fig. 2), we would expect about 21 predation events to plot in field II in Fig. 6B, rather than just two. The probability for the observed result to occur by chance was negligible (*p* < 10^–6^; binomial probability calculation). Finally, it was surprising that *O. semistriata* of over 19 mm shell length were hardly ever found in *A. propatula*’s pouches (field III in Fig. 6B), although large specimens of up to 22 mm were quite common at Playa Grande.

### Conspecific prey (cannibalism)

**Figure 7:**
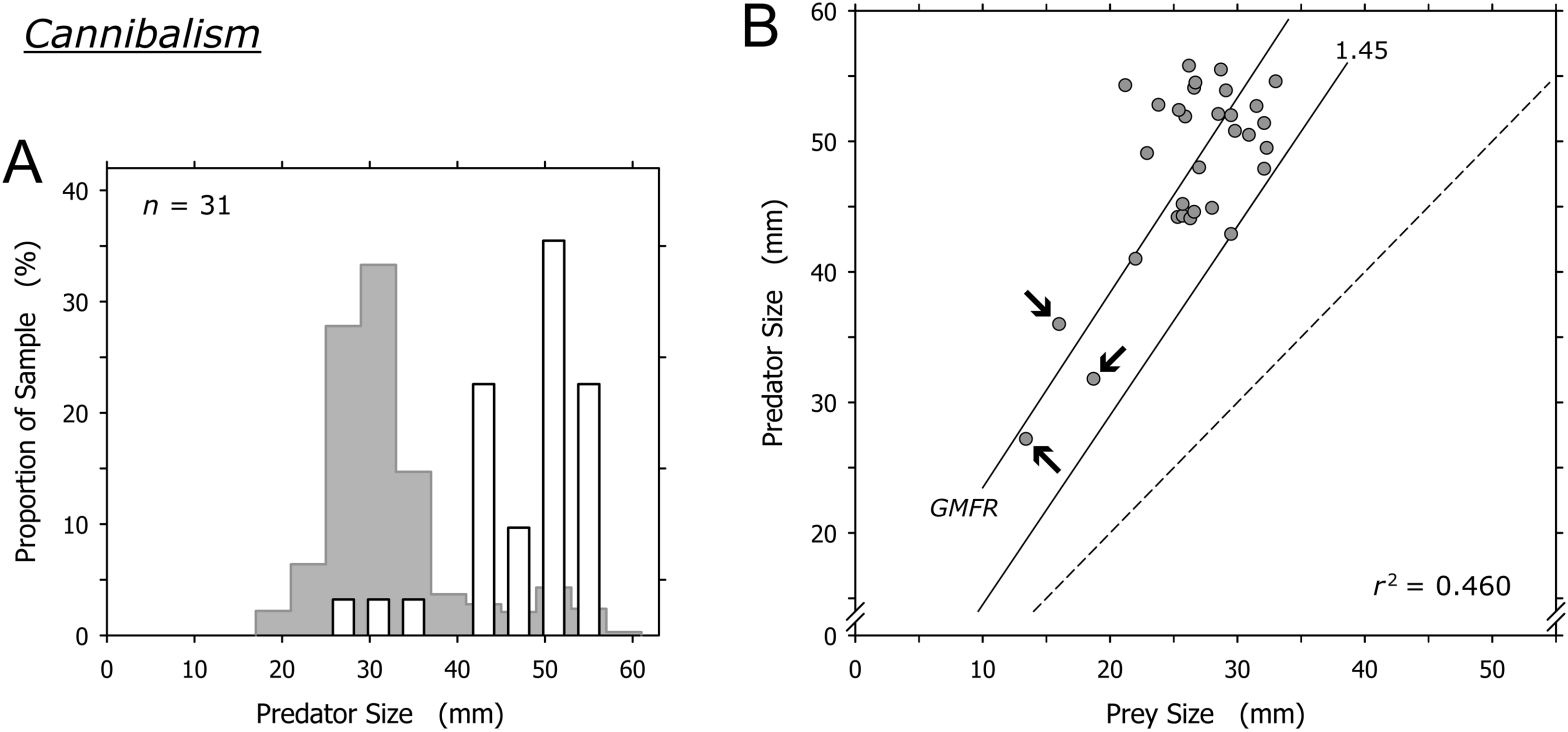
Relationship of predator and prey shell lengths in cannibalistic interactions in *Agaronia propatula*. (A) Size distribution of *A. propatula* carrying conspecifics in their metapodial pouches (white bars). In the background, the size distribution for all *A. propatula* carrying prey is shown. (B) Predator size plotted versus prey size. *r*^2^ and the geometric mean functional relationship (GMFR) are given, and the predator-to-prey size ratio of 1 (dashed line) as well as the postulated threshold ratio for gastropod predation (1.45) are marked. Arrows point at three outliers on which the correlation depended.

Cannibalism was conducted mainly by *A. propatula* over 41 mm, and only 3 of 31 cannibalization events were conducted by predators smaller than this threshold (Fig. 7A). The prey in these three events were among the smallest *A. propatula* observed in this study and ranged from 13.4 to 18.7 mm. There was a weak correlation between predator and prey size (*r*^2^ = 0.460; Fig. 7B), but this was strongly influenced by the three events involving small cannibals. When these three outliers were omitted from the analysis, no correlation was evident (*r*^2^ = 0.041). As was the case for heterospecific gastropod prey (Fig. 6B), successful cannibals were at least 1.45 times larger than their conspecific prey (Fig. 7B). We never observed autotomy in the victims of cannibalism.

## Discussion

By building on earlier studies on the foraging behavior of *A. propatula*, we were able to gain a more detailed understanding of the complex relationship between *A. propatula* and its prey. Our analysis of 327 predation events compiled over a six-year period at Playa Grande corroborates several earlier conclusions. (1) *A. propatula* preys on a variety of invertebrate taxa present in its sandy beach ecosystem, but there are notable exceptions such as echinoderms and worm-like animals (Table 1). While *A. propatula* actually avoids the sand dollars that share its habitat (Cyrus et al., 2012), nothing is known about its interactions with the annelids and flatworms on this beach. (2) *A. propatula* never collects more than one prey item in its pouch, in contrast to some other Olividae (Taylor & Glover, 2000; Kantor & Tursch, 2001). (3) *A. propatula* is capable of subduing bivalve and crustacean prey larger than itself (Figs. 4B and 5B), highlighting the species’ predatory prowess and the efficiency of its prey capturing mechanism involving the metapodial pouch (Cyrus et al., 2012). (4) The gastropod *O. semistriata* is the dominant prey species (Table 1). This may be explained by the sheer abundance of this species on Pacific Central American beaches rather than the prey preferences of *A. propatula* (Cyrus et al., 2012). (5) Cannibalism occurs at a significant frequency (Table 1) and therefore is an important factor in the trophic relationships of *A. propatula* (Cyrus et al., 2015). (6) *A. propatula* captures gastropods only if it is at least 1.45 times larger than its prey (Figs. 6B and 7B). Gastropods, unlike the much less mobile bivalves, therefore appear sufficiently agile to avoid predation by *A. propatula* if they are large enough relative to their potential predator (Cyrus et al., 2015). (7) Autotomy is not performed to defend against cannibalistic attacks (Cyrus & Peters, 2014; Cyrus et al., 2015).

Our study included measurements of individual predator and prey sizes, which provided additional insight into the trophic relationships between *A. propatula* and its prey. For example, we compared the size distribution of successful predators (animals with prey in their metapodial pouches; this study) to the previously determined size distribution of foraging *A. propatula* (animals actively crawling on the sediment; Cyrus et al., 2015) at the same location. Compared to the distribution of foraging animals, large specimens appeared over-represented and small ones under-represented in the distribution of successful predators (Fig. 2). One plausible interpretation of this finding is that predatory success is influenced by predator size. If smaller *A. propatula* have to forage for longer periods than larger specimens before capturing a prey item, the relative proportion of small animals will be lower among successful predators than among foraging animals. The opposite will be true for large animals. However, an alternative interpretation also is possible. The size distribution of successful predators (Fig. 2) was based on data collected over several years and therefore represents a long-term average, while the size distribution of foraging snails determined by Cyrus et al. (2015) represents a snapshot, as it was collected on a single day during the dry season. Much of the Pacific Central American coast experiences a pronounced contrast between a rainy and a dry season. Frequent torrential rains during the wet season could certainly induce severe hypo-osmotic stress for any osmo-conforming invertebrate that utilizes the intertidal zone at intermediate and low tide (for studies of the effect in other taxa, see Blockley et al., 2007; Morritt et al., 2007; Montory et al., 2014). *Mazatlania fulgurata*, a columbellid gastropod that shares the habitat with *A. propatula*, reproduces primarily in the dry season, which probably reduces the chance of exposure of eggs and larvae to hypo-osmotic stress (D. Stevenson & W. S. Peters, 2012–2018, unpublished data). If *A. propatula* has evolved similar seasonal adaptations, annual shifts in the size distributions of its populations must be expected, which could result in significant differences between the size distribution averaged over the year compared to the size distribution on any given day. Thus, we have two alternative hypotheses. The differences in the two size distributions compared in Fig. 2 may be due to a size dependence of predatory efficacy or to environmentally-induced seasonal variation in size structuring. To determine between the two, size distributions around the year as well as foraging efficiencies of different size classes of *A. propatula* will have to be determined.

Cyrus et al. (2012) stated that *A. propatula* is an opportunistic predator whose prey spectrum mirrors the availability of prey taxa at a given location. However, the absence of echinoderms and worms, which are common at Playa Grande, from the prey spectrum of *A. propatula* indicates limitations to the hypothesis. Our data instead indicate that the ecological function of *A. propatula* in its trophic network changes as it grows in size (Fig. 3), indicating ontogenetic niche shifts (Nakazawa, 2015) in a size-structured population (Polis, 1984; de Roos, Persson & McCauley, 2003; Rudolf, 2007). Yet the case is not as straightforward as the spectrum of potential prey simply broadening in parallel with *A. propatula*’s increase in size. For instance, there is no obvious reason why *O. semistriata*, the most abundant prey species overall (Table 1), should be such a rare find in metapodial pouches of predators exceeding 40 mm (Fig. 3). To clarify the situation, we analyzed the relationships between predator and prey size separately for each major prey taxon, and were thus able to assess how our data deviate from the hypothesis that *A. propatula* indiscriminately exploits the available spectrum of potential prey.

### Bivalve prey

Bivalves under 15 mm shell length were most commonly captured by intermediately sized *A. propatula* between 25 and 35 mm (Fig. 4B), which likely reflects the large proportion of this size group in the population of the predator. Larger *A. propatula* also caught small bivalves, and the largest predator in our sample, an animal of 57.4 mm, was found with a bivalve of only 9.6 mm in its pouch (Fig. 4B). On the other hand, with the exception of a single predation that was below the 1:1 predator-to-prey size ratio (right of the dashed line in Fig. 4B), *A. propatula* only captured bivalves when it was larger than the potential prey (data-points left of the dashed line in Fig. 4B). In other words, the size of the largest bivalves captured increased with predator size. We interpret this pattern as a predator size-dependent widening of the “predation window” (Claessen et al., 2002), characterized by an increase of the variance of prey size as the predator grows larger (Peters, 1983). This phenomenon occurs when the growth of a predator enables it to subdue increasingly large prey. If the growing predator also retains a capacity for capturing small prey—that is, if the maximum size of accessible prey increases with predator size more rapidly than minimum prey size does—predator and prey size will be correlated and the variance of prey size will increase with predator size. Predation window widening with increasing predator size is not uncommon (Scharf, Juanes & Rountree, 2000; Dörner & Wagner, 2003; Chabot et al., 2008; Polidori et al., 2009). The interaction between *A. propatula* and its bivalve prey (Fig. 4B) adds another example.

*Agaronia propatula* appears to exploit the entire size range of bivalve prey available at the study beach, since the largest bivalves (∼40 mm) found in metapodial pouches were similar in size to the largest individuals found free-living at the site over the six-year observation period. At the lower end of the bivalve prey size distribution, the sharp limit at 3.7 mm is intriguing (Fig. 4B, field I). Prey size can be a limiting factor for predation efficiency not only if the prey is too large to be subdued by a predator, but also if potential prey items are too small to be detected or handled efficiently (Claessen, de Roos & Persson, 2000; Claessen et al., 2002; Brose et al., 2008; Costa, 2009). We frequently observed small bivalves in metapodial pouches together with significant amounts of sand. This suggests that *A. propatula* can handle small prey more successfully if the item is taken together with some of the surrounding sediment (compare Cyrus et al., 2015). If so, completed but unsuccessful predation attempts may result in predators carrying pouches with sediment only, being unaware of the lack of edible victims in their catch. This probably explains the occasional occurrence of metapodial pouches filled with sand but without prey. The observed lower size limit of bivalve prey may therefore suggest that *A. propatula* are unable to handle prey of less than 4 mm efficiently. A problem for this interpretation is that the lower limit of prey size did not seem to correlate with predator size (Fig. 4B), which we would expect if mechanical difficulties in manipulating small objects were responsible for the limit. A more plausible explanation may instead be that the lower limit of the bivalve prey spectrum reflects the lower limit of the size distribution of the available bivalve prey.

Taken together, our results appear to support the view that *A. propatula* indiscriminately exploits the bivalve population in its habitat, limited only by decreasing success rates of attacks when the predator-to-prey size ratio approaches unity.

### Crustacean prey

There was no clear correlation between the size of *A. propatula* and their crustacean prey, and no unambiguous predation window widening (Fig. 5B). Most of the crustaceans found in metapodial pouches were dead or injured, and generally unable to move. This may explain the otherwise surprising fact that mole crabs and other crustaceans, which in a healthy state should be sufficiently agile to evade an approaching *A. propatula*, form a significant portion of the gastropod’s prey spectrum. In fact, small crustaceans frequently were seen scrambling from their hiding places under the sediment surface when an *A. propatula* drew close, moving rapidly out of the predator’s path and burrowing in again. Thus it seems that only dead, injured, or otherwise weakened crustaceans fall victim to *A. propatula*. This is in remarkable contrast to shelled prey, which in the majority of cases (gastropods: 90%; bivalves: 79%) resumed normal activities within 5 min of being released from metapodial pouches. Similarly, victims of cannibalistic attacks survived and retained locomotory capabilities when confined in pouches for many hours (Cyrus et al., 2015). We conclude that *A. propatula*, while being a predator of mollusks, acts as a facultative scavenger of crustacean prey.

Cyrus et al. (2012) put forward a similar argument regarding predation events involving very large *Emerita* sp. at El Cuco, El Salvador, suggesting that these mole crabs were immobile because they had recently molted. One may object that egg mass-carrying crustaceans—such as one of the *Emerita* sp. that we found in a pouch—are mature and therefore unlikely to just have undergone molting, but molt and reproductive cycles can alternate in short succession in the genus *Emerita* (Subramoniam & Gunamalai, 2003). Nonetheless, the argument by Cyrus et al. (2012) does not explain the lack of intermediately sized crustacean prey (field III in Fig. 5B) and the resulting isolated position of the cluster of events in which prey items were bigger than their predators (data-points right of the dashed line in Fig. 5B). We feel that the predatory interactions between *A. propatula* and *Emerita* sp. are too complex to be elucidated satisfactorily by data currently available.

### Gastropod prey (excluding cannibalism)

There was neither a correlation between the sizes of predators and *O. semistriata* (*r*^2^ = 0.081) nor an indication of predation window widening (Fig. 6B). The distribution of the data raised at least three questions. First, it remains unclear why *O. semistriata* of under 5 mm shell length never were found in pouches (field I in Fig. 6B). The developmental biology of the species including its life history as well as the size distribution of the population on the beach and its changes over the year will have to be determined to evaluate possible causes of this finding.

Second, the near complete absence of *O. semistriata* from the prey spectrum of *A. propatula* over 40 mm (field II in Fig. 6) cannot be explained by a lack of interest in this prey species from large *A. propatula*. In preliminary field experiments, we placed *O. semistriata* in the path of foraging *A. propatula* and found similar attack rates with predators of above and below 40 mm (S. D. Rupert & W. S. Peters, 2016–2018, unpublished data). We suggest an alternative explanation that is related to the tidal migrations both species perform by passive locomotion with the waves (Troost et al., 2012; Cyrus et al., 2012). Large *A. propatula* are most frequently found in the lowest part of the intertidal zone (A. Z. Cyrus, J. Swiggs & W. S. Peters, 2011–2018, unpublished observation), suggesting ontogenetic modifications of the species’ tidal migration behavior. If large predators remain confined to a zone around the low water line throughout the tidal cycle, they may rarely interact with *O. semistriata*, which need to follow the moving backwash zone to perform suspension feeding (Troost et al., 2012). If this is correct, we expect to find a dynamic separation zone between *O. semistriata* and large *A. propatula* that shifts constantly along the beach slope with the tides. Testing the hypothesis will require establishing size profiles of *A. propatula* along the beach slope at high temporal resolution.

Third, there was a threshold of the predator-to-prey size ratio for successful predation on gastropods, as suggested by Cyrus et al. (2015): *A. propatula* had to be at least 1.45 times larger (regarding shell length) than its gastropod prey for successful capture (Figs. 6B and 7B). However, our data indicate that the trophic relationship between *A. propatula* and *O. semistriata* is influenced by an additional size-dependent factor. With a size ratio threshold of 1.45, more than half of the *A. propatula* in our sample of successful predators (Fig. 2) should be capable of subduing *O. semistriata* of 19–22 mm shell length, a common size class at the study site. Therefore we should expect numerous data-points in field III in Fig. 6B. Intriguingly, this field is almost empty. This surprising finding is unlikely to be caused by a differential distribution of large *O. semistriata* (>19 mm) and foraging, appropriately sized *A. propatula* (>30 mm) on the beach, as we routinely observed them together in close vicinity. It rather seems that *O. semistriata* can outgrow the predation pressure exerted by even the largest *A. propatula*, independently of the predator-to-prey size ratio. This could be explained if the efficacy of flight responses in *O. semistriata* depended on body size in a non-linear manner. The validity of this hypothesis will have to be tested in the field by measurements of the velocities of differently sized animals in various predation-related situations.

### Conspecific prey (cannibalism)

Cyrus et al. (2015) concluded that cannibalism is a significant factor in the population ecology of *A. propatula*. Our enlarged dataset even points to a higher proportion of cannibalism in the prey spectrum (9.5% of all documented predation events; Table 1) than that reported by Cyrus et al. (5.6%).

In many but not all cannibalistic taxa, cannibalism is size-dependent with larger individuals preying on smaller ones (Polis, 1981). Among molluscs, this holds true for cephalopods (Ibáñez & Keyl, 2010) and shelled but not necessarily unshelled gastropods (Baur, 1992). Results from field experiments in which the size-dependence of the success rate of cannibalistic aggression in *A. propatula* was characterized, suggested three simple rules (Cyrus et al., 2015). First, cannibalistic attacks will be successful if the size ratio, expressed as shell length of the predator divided by the shell length of the victim, is at least 1.46. Second, the attacks will be unsuccessful if the ratio is less than 1.18. Third, if the ratio is intermediate (i.e., at least 1.18 but below 1.46), half of the attacks will be successful. Applying these rules, Cyrus et al. (2015) estimated the decline of intraspecific predation pressure a snail experiences as it is growing, demonstrating a selective pressure favoring rapid development.

**Figure 8:**
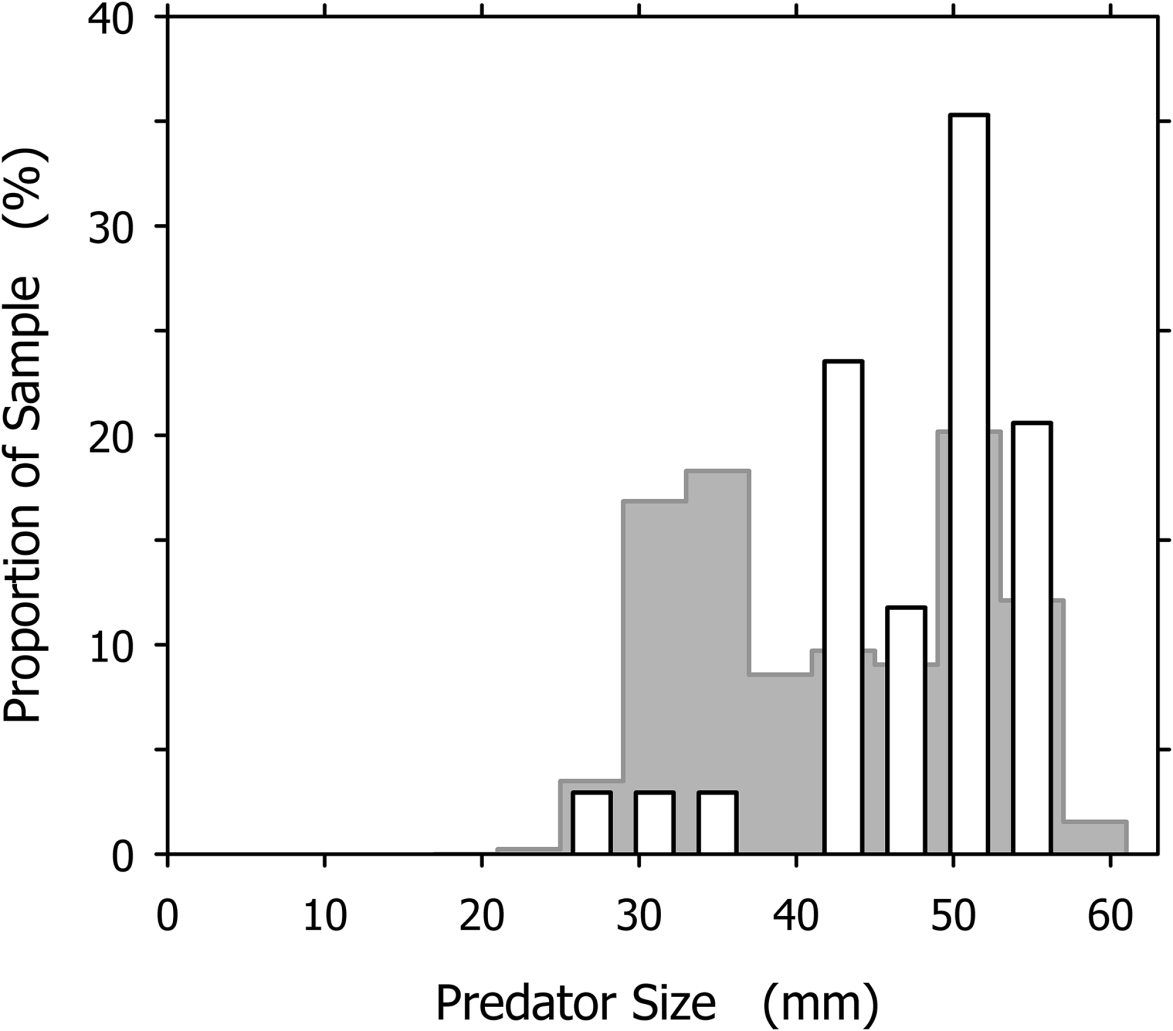
Comparison of the expected and observed distributions of successful cannibalistic predators across the size classes of *Agaronia propatula*. Gray bars represent the expected distribution, calculated assuming that individual *A. propatula* meet randomly on the beach, and given the size distribution of all successful predators (see Fig. 2) and the size-dependence of success in cannibalism determined by Cyrus et al. (2015). White bars show the observed distribution of successful cannibals (compare Fig. 7A).

It is tempting to interpret the dominance of large individuals among the successful cannibals (particularly conspicuous in Fig. 7A) as an effect of this size-dependence: larger snails are more efficient cannibals after all, so they must be over-represented in the group of successful cannibals. Our data, however, indicate an influence of additional factor(s). If we consider a group of *A. propatula* with the size distribution of our sample of 327 successful predators (Fig. 2; see original data in Table S1), and further assume that these animals meet randomly on a beach (i.e., the probabilities for a given snail to encounter one of its 326 conspecifics are identical for all possible pairings), then we can apply the three rules of Cyrus et al. (2015) to generate the expected size distribution of cannibals in our sample (for details, see Table S2). This expected distribution exhibits a bimodal shape with two peaks (Fig. 8, gray bars). The peak in the large size classes is caused by the high success rate of big animals in cannibalistic interactions—large specimens are a small minority in our sample (compare Fig. 2), so conspecifics they encounter are almost always much smaller and therefore subdued easily. The peak at intermediate sizes (above 30 mm shell length) is caused by the large proportion of intermediately sized animals in our sample. While their success rate in cannibalism is low, their number is high, so that they appear prominently in the expected size distribution of successful cannibals.

The expected size distribution differs remarkably from the observed distribution, which for comparison is also shown in Fig. 8 (white bars). Compared to expectations, individuals of less than 40 mm shell length are under-represented while those over 40 mm are over-represented among the observed cannibals. The difference between the expected and the observed distributions (Fig. 8) is statistically significant at *p* < 0.01 (*χ*^2^ = 22.191, d*f* = 10). This finding is unlikely to result from an over-estimation of the intraspecific aggressiveness of small (<40 mm) *A. propatula* in our model calculations, as the behavioral field experiments by Cyrus et al. (2015) compellingly demonstrated that animals of all sizes will be cannibals if they can. The proportional mismatch between the expected and the observed size distributions (Fig. 8) could be explained more convincingly if our assumption of random encounters between the individuals were incorrect. Above we had suggested that certain features of the predator–prey size relationship of *A. propatula* and *O. semistriata* could be explained by ontogenetic modifications of the tidal migration behavior of *A. propatula*. We now hypothesize that the resulting differential distribution of the size classes of *A. propatula* along the beach slope also affects the encounter rates between the size classes, and thus the frequency of cannibalism in the different size classes. An accurate characterization of the localization of animals of different sizes throughout the tidal cycle will be required to develop this idea further.

## Conclusion

In summary of our findings we conclude that by increasing the breadth of our database to include size measurements of individual predators and their prey, the identification of unexpected prey taxon-specific and predator size-dependent predation patterns has been facilitated. These patterns document a previously unknown complexity of the trophic relationships of *A. propatula*. At various points in our above discussion, hypotheses concerning the behavioral mechanisms that cause the observed patterns have emerged. These hypotheses are testable and will guide future studies.

## Supplemental Information

10.7717/peerj.4714/supp-1 Supplemental Information 1 Original raw data including date, location, prey taxon, and predator and prey sizes for each recorded predation event.

10.7717/peerj.4714/supp-2 Supplemental Information 2 Calculation of the expected size distribution of successful predators.

## Additional Information and Declarations

### Data Availability

The following information was supplied regarding data availability: The original data describing each individual predation event are available in Table S1.

### Field Study Permissions

The following information was supplied relating to field study approvals (i.e., approving body and any reference numbers): This study was performed under the research permits ACT-OR-D-015 and ACT-OR-DR-064 to W.S.P. from the Costa Rican Ministerio de Ambiente y Energia.
